# Sex-Related Differences in Drugs with Anti-Inflammatory Properties

**DOI:** 10.3390/jcm10071441

**Published:** 2021-04-01

**Authors:** André Farkouh, Christoph Baumgärtel, Roman Gottardi, Margit Hemetsberger, Martin Czejka, Alexandra Kautzky-Willer

**Affiliations:** 1 Department of Pharmaceutical Sciences, University of Vienna, 1090 Vienna, Austria; martin.czejka@univie.ac.at; 2 AGES Austrian Medicines and Medical Devices Agency and Austrian Federal Office for Safety in Health Care, 1200 Vienna, Austria; christoph.baumgaertel@ages.at; 3 Vascular Surgery, MediClin Heart Institute Lahr/Baden, 77933 Lahr, Germany; roman.gottardi@gmail.com; 4 Hemetsberger Medical Services, 1070 Vienna, Austria; contact@hemetsberger.at; 5 Gender Medicine Unit, Division of Endocrinology and Metabolism, Department of Internal Medicine III, Medical University of Vienna, 1090 Vienna, Austria; alexandra.kautzky-willer@meduniwien.ac.at

**Keywords:** sex, gender, NSAID, anti-inflammatory drug, pharmacokinetics, pharmacodynamics, steroid, SARS-CoV-2, COVID-19

## Abstract

There is increasing evidence of sex differences in the action of anti-inflammatory drugs, with women being at significantly higher risk of adverse effects. Nevertheless, clinicians’ awareness of the implications of these sex differences on dosing and adverse event monitoring in routine practice is still in need of improvement. We reviewed the literature evaluating sex differences in terms of pharmacokinetics and pharmacodynamics of anti-inflammatory drugs. The anti-thrombotic activity of selective and non-selective COX-inhibitors tends to be stronger in men than women. Side effect profiles differ with regards to gastro-intestinal, renal and hepatic complications. Glucocorticosteroids were found to be more effective in men; women were more sensitive to corticosteroids when their oestradiol levels were high, a finding important for women taking hormonal contraception. TNF-alpha inhibitors have a longer half-life in men, leading to stronger immunosuppression and this a higher incidence of infections as side effects. Although research on sex differences in the effectiveness and safety of drugs is increasing, findings are often anecdotal and controversial. There is no systematic sex-differentiated reporting from clinical trials, and women are often under-represented. As personalized medicine is gaining in importance, sex, and gender aspects need to become integral parts of future research and policy making.

## 1. Introduction

Numerous investigations have documented that women, in general, are dispensed more prescription and over-the-counter (OTC) drugs compared to men [1]. Gender-specific differences may play an important role in pharmacotherapy, which was long underestimated. Furthermore, incidences of several diseases differ between men and women. For instance, women suffer more often and more severely from osteoporosis [2], asthma [3], migraines [4], depression [5], irritable bowel diseases [6], or autoimmune diseases such as rheumatoid arthritis, lupus erythematosus, or multiple sclerosis [7,8]. In contrast, men are more likely to suffer from various forms of cancer e.g., hepatocellular carcinoma [9] or cardiovascular diseases [10]. In this context, it must be noted that women typically display a later onset of coronary heart disease than men [10]. This may be related to cardioprotective effects of oestrogen [10,11].

In the past, research has focused on the male organism, and women tended to be under-represented in clinical trials, as were female rodent models from pre-clinical research [12,13,14,15]. However, an increasing number of studies have shown sex-specific differences in the effects of drugs which, while mostly pharmacokinetic in nature, can also be caused by pharmacodynamic differences [14,16]. There are also trivial aspects that play a partly decisive role: on average, women have lower body height and weight than men so that a given drug dose leads to a higher concentration of the active ingredient in women. Women have a higher body-fat ratio while their body-water ratio is lower, which has major effects on the concentration, distribution, and effect duration of drugs [16,17]. Hence, lipophilic agents have a higher volume of distribution (Vd) in women, while hydrophilic active ingredients have a lower Vd. Therefore, identical doses of a lipophilic drug led to lower plasma concentrations in women than men, while a hydrophilic one leads to higher plasma concentrations [16]. 

There are also remarkable differences between the sexes regarding the activity of drug metabolizing enzymes, both in phase I (drug functionalization) and phase II (drug conjugation). Additionally, some efflux transporters like P-glycoprotein (P-gp, ABCB1) and the Breast Cancer Resistance Protein (BCRP) are more active in men than in women [18,19,20,21]. Potential mechanisms behind the differential functioning of male and female bodies have been reviewed extensively elsewhere [15,21,22,23].

Irrespective of the known sex disparities, a uniform pharmacological treatment approach has been followed for men and women, most notably, women are treated with drug doses derived from studies carried out on men. Hence, women suffer twice as often from drug-induced side effects, e.g., drug-induced long QT syndrome [24].

## 2. Materials and Methods

This review focuses on inflammation and describes important examples of anti-inflammatory drugs, including some OTC drugs, with substantial and clinically relevant sex-related differences in their pharmacokinetic and pharmacodynamic properties. It also discusses the consequences of these disparities for prescription and use of such drugs. However, important topics such as sociocultural gender and biomarkers were not addressed. PubMed (incl. PubMed Central and Medline) was searched using the following search terms and their combinations: “gender”, “differences”, “pharmacokinetics”, “pharmacodynamics”, “NSAID”, “steroid”, and “anti-inflammatory”. Additionally, reference lists of identified articles and key systematic reviews were searched manually. Relevant articles in English and German published between 1985 and 2021 were considered.

## 3. Sex-Specific Differences in Immune Responses and Inflammatory Diseases

The body’s immune response, i.e., the body’s protection and defence system against both its own and foreign antigens, is substantially different between the sexes. In general, the body at first responds to tissue damage or pathogen intrusion (e.g., virus, bacteria, fungal infection or parasites) with an acute inflammation. These inflammatory processes serve to mitigate the harmful influence, and to initiate healing and regeneration processes. However, if this fine-tuned cascade spirals out of control, the inflammation cannot be stopped and leads to chronic inflammatory disease [25].

**Figure 1:**
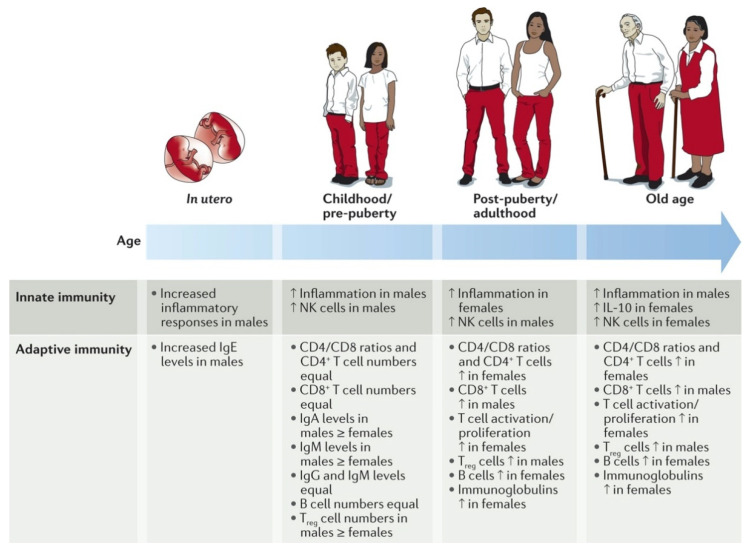
Gender-specific differences in immune responses and inflammatory diseases. Multiple immunological factors vary between the sexes throughout the course of life. For certain factors (for example, pro-inflammatory responses), the sex differences change at puberty and then wane in later life, suggesting hormonal effects. For other factors, the sex difference remains constant from birth to old age (for example, higher numbers of CD4^+^ T cells and CD4/CD8 T cell ratios in females). The paucity of studies in this area is notable, particularly in utero sex differences in which results are conflicting. IL-10, interleukin-10; NK, natural killer; T_reg_, regulatory T. Reproduced with permission from [26].

As reviewed extensively by Klein et al., numerous studies have shown remarkable sex disparities regarding the activities of the innate and adaptive immune systems (Figure 1) [26]. 

It could be shown, for instance, that elderly women have a more active innate and adaptive immune system than men of the same age [27]. However, age seems to be a key factor as the immune responses and inflammation activities are more pronounced in boys up to puberty but afterwards, are more pronounced in women [28].

Research of the underlying mechanisms has shown very complex differences in numerous immune cells driving the innate and the adaptive immune responses. For innate immunity, it could be shown that the phagocytosis by neutrophils and macrophages, the macrophages’ activity, the type-1 interferon (IFN) activity of the dendritic cells, and the efficiency of the antigen-presenting cells are all more pronounced in female cells than in their male counterparts (Table 1) [7,26].

In contrast, men have higher numbers of natural killer (NK) cells and the expression of toll-like receptors (TLR) on macrophages and neutrophils. With regard to acquired immunity, women have more B-lymphocytes and, therefore, better antibody production, higher amounts of active T-lymphocytes with higher proliferation rates, more CD4+ cells, and a pronounced T-cell cytotoxicity. Men, on the other hand, have higher concentrations of regulated T-lymphocytes and CD8+ cells [26].

Due to the more active immune system in women, it is obvious that their inflammatory response will be stronger than mens. Various pro-inflammatory processes, e.g., the release of the immune-stimulating IFN-γ and interleukin (IL)-17 by T-lymphocytes or the plasma levels of cytokines such as tumour necrosis factor (TNF) and IL-6, are more pronounced in women [26]. On the other hand, the release of the inflammation-inhibiting cytokines IL-4 and IL-10 following the leukocyte stimulation are more pronounced in men.

Differences in male and female immune responses were also observed in patients who had acquired SARS-CoV-2 [29]. Male sex is associated with more hospitalizations, more severe course of disease, and higher mortality [30]. Studies found lower T-cell frequencies and activation in men [31,32], downregulation of B-cell activity and NK cell activating receptors in men [32], as well as a predominance of men among patients with neutralizing IgG autoantibodies against IFN-ω and/or IFN-α, enabling easier SARS-CoV-2 infections [33,34,35]. Sex differences in immune responses have been shown to cause differences in vaccine effectiveness [35,36]. Interestingly, analyses for sex differences are mostly lacking or of a rudimentary level in reports of trials investigating antiviral drugs, corticosteroids, and–importantly–vaccines [35,36,37].

Sex discrepancies between the innate and acquired immune responses are not only caused by their sex chromosomes but also by differences in sexual hormones [38]. Many genes on the female X-chromosome (e.g., genes for TLR) regulate immune function and contribute to the predominance of autoimmune diseases in women [26,38]. Polymorphisms of the male Y-chromosome contribute to the higher susceptibility to viral infections. The so-called Klinefelter syndrome in men is caused by an extra X-chromosome and is associated with a reduced testosterone level and increased oestrogen levels [39]. Men with Klinefelter syndrome develop autoimmune diseases more often, and their immune response is similar to that of women. For example, the immunoglobulin concentration and numbers of B-cells and CD4+-T-cells in men with Klinefelter syndrome are elevated but can be reduced through testosterone therapy [40].

Oestradiol stimulates the inflammation process in accordance with the higher inflammation activity of the female immune cells, while testosterone acts on inflammation inhibitors [38,41]. Half of the genes in activated T-cells have response elements for oestrogen receptors. Testosterone is generally classified as antinociceptive while oestradiol and progesterone can act as a pro- as well as an antinociceptive agents. In experiments using castrated rats, oestradiol substitution reduced the pain threshold while testosterone substitution increased it [42]. Interestingly, high oestradiol levels in patients with rheumatoid arthritis suppressed the pain threshold while lower concentrations stimulated it [43]. Oestradiol increases the formation of immunoglobulin, the release of IFN-γ from leukocytes and the activity of nuclear factor kappa-light-chain-enhancer of activated B cells (NF-κB) in T-lymphocytes [26,43]. Testosterone reduces elevated biosynthetic pro-inflammatory leukotriene levels in granulocytes and monocytes, and high testosterone plasma levels correlate with lower leukotriene formation in women [44]. 

In addition to influencing the pathophysiology of inflammation, sex hormones affect the pharmacodynamics and pharmacokinetics of drugs [21]. For example, oestradiol slows gastric emptying, increases the body-fat ratio, and reduces the amount of α1-glycoprotein, which non-specifically binds alkaline drugs. Furthermore, sex hormones such as oestrogen (primarily as oestradiol) in women and androgens (primarily testosterone) in men are directly or indirectly involved in many transmembrane transport processes [21].

Taken together, these findings have profound consequences on the defence mechanisms against infections, autoimmune diseases, malignant diseases, and on vaccinations.

## 4. Drugs with Anti-Inflammatory Properties

Due to the fact that inflammatory diseases predominate in women, they use anti-inflammatory drugs and painkillers much more often [45].

### 4.1. Non-Steroidal Anti-Inflammatory Drugs (NSAIDs)

NSAIDs are the most frequently used anti-inflammatory drugs. They suppress the binding of pro-inflammatory prostaglandins and thromboxane by non-specific inhibition of cyclooxygenase (COX)-1 and COX-2. Age and sex-specific disparities regarding side effects of various COX-1/2 non-selective NSAIDs have been identified [46]. For example, men experience more frequent serious gastrointestinal events than women [46,47]. In a case control study conducted in a French pharmacovigilance database, a direct correlation between NSAID exposure and liver injury was found in women but not in men [48]. In a case control study of 88 cases and 178 controls, a significant association was found between liver injury and NSAID exposure in women (odds ratio [OR] = 6.49 [95% CI 1.67–25.16]) but not in men [OR = 1.06 (0.36–3.12)] [49]. It has been hypothesized that this may be due to different pharmacokinetics or circulating hormone levels, polypharmacy in women or generally higher risk of drug-induced liver injury in women [48,50]. However, a similar risk of NSAID-induced acute liver injury for men and women and younger versus elderly participants was found in a retrospective cohort study [51]. Additionally, no difference between men and women using NSAIDs was found for the risk of Parkinson’s disease [52].

#### 4.1.1. Acetylsalicylic Acid (ASA, Aspirin)

ASA has a special place amongst the NSAIDs because it irreversibly inhibits prostaglandin and thromboxane production by the COX-1 enzyme. Moreover, through acetylation of COX-2, it causes the formation of anti-inflammatory lipoxins (so-called ASA-triggered 15-epi-lipoxin, ATL) [53], which are much more predominant in women than men [54]. In addition to often being used for treating fevers, mild to medium pain, and inflammation, it is also used as thrombosis prophylaxis. 

Due to its efficacy in inhibiting the formation of thromboxane A2 in platelets, ASA is a strong antiplatelet agent. It is also well established in the secondary prevention of cardiovascular diseases with similar benefits for both men and women. Its use in primary prevention is controversially discussed owing to contradictory results between the sexes [21,44]. Extensive studies have shown that low dose ASA (100–300 mg) protects men from myocardial infarction but not from ischemic stroke, with opposing findings in women [55,56]. Aggregation studies using female platelets found lower effectiveness of ASA to inhibit platelet aggregation, which may be due to the influence of female sex hormones [57].

However, these observations from clinical practice cannot be accurately understood using pharmacokinetic models, because the resorption rate for ASA varies greatly between individuals and depends, among other factors, on the filling state of the stomach and on the gastric pH, with better resorption at low pH [21,58]. Since women have a slightly higher gastric pH, a lower absorption would be expected. On the other hand, since the activity of the glucuronidating enzymes is lower in women, the excretion of absorbed ASA is retarded by 30–40% [59].

A retrospective review of patients using drugs interfering with the defence mechanism of the gastrointestinal mucosa, including ASA, warfarin, NSAIDs, or selective serotonin re-uptake inhibitors (SSRIs), reported a male preponderance for upper gastrointestinal bleeding and an equal sex distribution among lower gastrointestinal bleeding [60]. In contrast, women taking low-dose ASA regularly, especially at age greater than 70 years, experienced an increased risk of gastrointestinal complications [59,61].

#### 4.1.2. Ibuprofen

Ibuprofen, another commonly used drug in the NSAID class, has a high therapeutic importance especially in the treatment of mild to moderate pain and fever. After rapid oral absorption the area under the plasma concentration-time curve (AUC) is dose-dependent, with extensive binding to plasma albumin [21,62]. 

Apparently, the analgesic effect predominates in men. Experimental studies have shown that men, who have a higher threshold and greater tolerance for electrically induced pain, responded significantly to ibuprofen, while women did not [63,64]. In other experiments, however, the pain lowering effect of ibuprofen was comparable in men and women [65]. Pharmacokinetic discrepancies for ibuprofen between men and women have not been observed. Therefore, the alleged better analgesic effect in men remains unclear. It is hypothesized that the difference in study outcomes is due to differences in the type of pain receptors involved in experimental pain models as opposed to pain experienced in clinical practice and also to nociceptive mechanisms. The potential sex difference in nociception might be connected to oestrogen effects on the activity of the nervous system, resulting in an improved transmission of pain impulses [21,64,66].

Furthermore, the results of one study highlighted that elderly males and those with coronary artery disease were at greater risk of ibuprofen-induced renal impairment compared to acetaminophen recipients [67]. Renal function should thus be monitored in these high-risk patients, especially in long-term users.

#### 4.1.3. Diclofenac

Several published reports found greater susceptibility for diclofenac-related liver injury among women than men [49,68,69,70,71]. In an analysis of 180 cases of hepatic injury associated with diclofenac, female patients were at significantly higher risk of injury (relative risk 2.0; *p* < 0.001) [68]. However, currently available evidence concerning differences between men and women is limited and does not support differentiation in dosing or treatment [72].

#### 4.1.4. Naproxen

One investigation on osteoarthritis patients found significantly higher plasma concentrations of naproxen in women than men, increasing proportionately with womens age [20,73]. However, no association between increased plasma concentrations and adverse events or efficacy was reported [73]. Studies in rats showed higher unbound interstitial fluid concentrations and lower 50% inhibitory concentration (IC_50_) values for naproxen in male animals, leading to overall stronger effects [74].

### 4.2. Selective COX-2 Inhibitors-Coxibs

Selective COX-2 inhibitors are associated less with gastrointestinal complications and abdominal symptoms than non-selective COX inhibitors.

Surveys have shown that 85% of COX-2-selective inhibitors are prescribed to women [75]. Paradoxically, sex-specific data from clinical trials are underreported. One analysis reports that 80% of the investigated rofecoxib trials did not describe efficacy results by sex and only one trial reported side effects by sex [76]. An Italian study found a higher risk of adverse reactions to COX-2 selective inhibitors in women than men [77].

Etoricoxib, a selective COX-2 inhibitor and a member of the bipyridine class, is available in numerous countries in Europe. The current scientific evidence shows a clear underrepresentation of women in published etoricoxib trials, specifically in phase I studies. Sex-stratified data on efficacy and adverse effects are scarce in these investigations. Nevertheless, a comparative study reported a higher risk of thrombosis in women using etoricoxib [78].

A study investigated vasoregulation in young patients with type 1 diabetes, hypothesizing that COX-2 inhibition would be associated with preferential vasoconstriction in women and would augment their response to angiotensin II [79]. In this study, maintenance of normal renal and peripheral blood vessel function was more dependent on vasodilatory prostaglandins such as angiotensin II in women compared with men. COX-2 inhibition eliminated this sex difference.

### 4.3. Glucocorticoids

Synthetic glucocorticoids are used as anti-inflammatory agents in an effort to mimic the role of their natural counterparts as primary mammalian anti-inflammatory hormones. A study in rat liver tissue, a classic glucocorticoid-responsive organ, explored sexually dimorphic actions of glucocorticoid regulation of gene expression. A comparison of the number of genes involved in inflammatory disorders revealed 84 additional glucocorticoid-responsive genes in male rats, suggesting that glucocorticoids are more effective in males. A sepsis model of systemic inflammation substantiated these gender-specific actions of glucocorticoids in vivo [80].

In humans, sex differences in the metabolism of cortisol have been reported [81,82]. One investigation showed considerable sex differences in both the production and metabolism of cortisol. In particular, a sex difference in 11 beta-hydroxysteroid dehydrogenase (11-HSD) activity was identified, with relatively greater conversion of cortisol to cortisone in women [82]. Cortisol and testosterone secretion patterns were found to be associated with variations in the incidence of cardiovascular disease, type 2 diabetes, and hypertension in men [83].

#### 4.3.1. Prednisone

Prednisone is commonly used in the treatment of myasthenia gravis, with highly variable dosages and dosing frequencies reflecting the absence of a standard protocol. Intolerable adverse effects were more commonly reported among women, leading to an unwillingness to accept a dose increase [84].

#### 4.3.2. Methylprednisolone

Women are more sensitive to methylprednisolone: they eliminate the drug more quickly, showing a greater clearance and a shorter elimination half-life. Consequently, a significantly smaller IC_50_ value for suppression of cortisol secretion was observed in women [85]. The IC_50_ values for effects of methylprednisolone on basophil trafficking related to oestradiol concentrations in women, with increased sensitivity found at higher oestradiol concentrations—a finding particularly important in the view of the number of women worldwide taking hormonal contraception [85,86]. It was suggested, however, that women’s increased sensitivity to methylprednisolone was balanced by their quicker drug elimination [85].

#### 4.3.3. Hydrocortisone

A study investigated the pharmacokinetics of hydrocortisone in pubertal patients with congenital adrenal hyperplasia [87]. When corrected for body mass index (BMI), cortisol clearance was higher, and the volume of distribution was greater in males than females. There was no difference in the half-life of total cortisol between the sexes. Free cortisol clearance did not differ significantly between the sexes, however, the BMI-corrected volume of distribution was higher in males and the half-life was significantly shorter in females [87].

### 4.4. TNF-Alpha Inhibitors

Etanercept was the first tumour necrosis factor (TNF) inhibitor approved by the FDA for the treatment of rheumatoid arthritis. Today these medications are used worldwide to treat autoimmune diseases such as Bechterew’s disease, rheumatoid arthritis, juvenile and psoriatic arthritis, inflammatory bowel disease (Crohn’s and ulcerative colitis), and psoriasis. Presently, two principles of TNF-alpha blockade are available: Etanercept is a soluble TNF receptor, while infliximab, adalimumab and golimumab are monoclonal antibodies against TNF; certolizumab is a pegylated antibody.

It is assumed that the androgens in the synovial tissue of men enhance the effect of TNF blockers, e.g., in Bechterew’s disease [75]. TNF inhibitors also have a longer half-life in men. However, blocking TNF causes immune suppression and, thus, a higher risk of developing serious infections [88]. Men more frequently experience infections as side-effects of TNF inhibitors than women, however, men with Bechterew’s disease on average show longer treatment adherence than women. The most frequent TNF inhibitor side-effects observed in women are toxic liver disease and lupus-like syndrome [89,90]. Moreover, the female sex has been associated with more severe adverse reactions to anti-TNF antibody treatment in patients with inflammatory bowel disease and paediatric Crohn’s disease [91,92,93].

### 4.5. Antileukotrienes

Antileukotrienes like the receptor antagonist montelukast act by inhibiting the lipoxygenase pathway, which is involved in the formation of pro-inflammatory leukotrienes from arachidonic acid. Therapies with these agents reduce exercise-induced asthma and have a modest suppressive effect on asthma symptoms.

Age and sex differences in montelukast efficacy were observed. In a randomized, controlled trial, boys aged 2 to 5 years showed greater benefit than did older boys, whereas among girls, the treatment effect was most evident in 10- to 14-year-olds. Moreover, non-significant effects were observed in younger girls [94].

Furthermore, examinations show that leukocytes such as neutrophils, monocytes and macrophages produce considerably lower amounts of leukotrienes in the 5-lipoxygenase pathway in men than in women [95,96,97]. The suppressive effect of endogenous testosterone in men, which causes changes in the localization of the key enzyme 5-lipoxygenase in leukocytes, might explain this observation [96,97].

## 5. Summary and Conclusions

Sex is an important factor in epidemiology, pathophysiology, clinical manifestations, disease progression, and response to treatment [22]. We have not addressed the equally important aspect of sociocultural gender, which has recently been reviewed elsewhere [22,23,98]. The field of sex and gender research is slowly gaining speed, but fundamental evidence gaps still need to be addressed to better understand the way these characteristics function independently and together to influence health, disease, and health care [22,23]. Importantly, more work is needed to standardize the way sex and gender are reported in clinical research and adequate transparency needs to be demanded by editorial policies and regulators [98,99,100]. Ideally, demographics, disease characteristics, and all outcome data should be disaggregated by sex, gender, or both, and studies should be powered adequately [101,102]. Clinicians’ awareness of sex- and gender-informed decision-making must be raised through appropriate continuous medical education, and routinely be incorporated into clinical decision-making [22,23].

In summary, research has demonstrated the importance of biological differences between men and women due to genetics, genome regulation, epigenetics, endogenous factors, and sociocultural, exogenous factors in variations seen in the safety and efficacy of drugs as well as the incidence, presentation, and diagnosis of diseases. There are ever-increasing efforts to identify biomarkers to individualize diagnosis and treatment, but often neglecting sex and gender. Sex and gender are an integral part of the personalized approach and need to gain importance in future research efforts, medical education, and policy making.

## Figures and Tables

**Table 1:** Sex differences in innate and adaptive immune responses in adults *.

Immune Component	Characteristic	Sex Difference
*Sex differences in the innate immune system*		
TLR pathways	TLR pathway gene expression	Higher in females
	TLR7 expression	Higher in females
	IL-10 production by TLR9-stimulated PBMCs	Higher in males
APCs	APC efficiency	Higher in females
Dendritic cells	TLR7 activity	Higher in females
	Type 1 interferon activity	Higher in females
Macrophages	TLR4 expression	Higher in males
	Activation	Higher in females
	Phagocytic capacity	Higher in females
	Pro-inflammatory cytokine production	Higher in males
	IL-10 production	Higher in females
Neutrophils	Phagocytic capacity	Higher in females
	TLR expression	Higher in males
NK cells	NK cell numbers	Higher in males
*Sex differences in the adaptive immune system*		
Thymus	Size of thymus	Larger in males
T cells	CD4^+^ T cell counts	Higher in females
	CD4/CD8 T cell ratio	Higher in females
	CD8^+^ T cell counts	Higher in males
	Number of activated T cells	Higher in females
	T cell proliferation	Greater in females
	Cytotoxic T cells	Increased cytotoxic activity in females
	T_H_1 versus T_H_2 cell bias	T_H_2 cell bias in females, T_H_1 cell bias in males
	T_reg_ cell numbers	Increased in males
B cells	B cell numbers	Increased in females
Immunoglobulins	Antibody production	Higher in females

APC, antigen-presenting cell; IL, interleukin; NK, natural killer; PBMCs, peripheral blood mononuclear cells; TH, T helper; TLR, Toll-like receptor; T_reg_, regulatory T. * Based on data from humans and rodents and primary cell cultures. Reproduced with permission from [26].

## Data Availability

Not applicable.
